# The Role of Dynamic Risk and Protective Factors in Predicting Violent Recidivism: Intellectual Ability as a Possible Moderator?

**DOI:** 10.1177/0306624X221079695

**Published:** 2022-02-28

**Authors:** Karolien Garritsen, Marija Janković, Erik Masthoff, Elien De Caluwé, Stefan Bogaerts

**Affiliations:** 1 Tilburg University, The Netherlands; 2 Fivoor Science and Treatment Innovation (FARID), Rotterdam, The Netherlands

**Keywords:** Historical-Clinical-Future Revised, dynamic risk factors, dynamic protective factors, violent recidivism, intellectual ability

## Abstract

This study investigated which risk and protective factors, based on the 14 clinical indicators of the Historical-Clinical-Future Revised, significantly predicted violent recidivism in a sample of 315 male forensic psychiatric patients. Additionally, it was investigated whether these associations were moderated by intellectual ability. Regarding risk factors, a stronger influence of risky network members, and higher levels of hostility, impulsivity, and addiction significantly predicted violent recidivism. Likewise, regarding protective factors, poorer social and labor skills, and a lower degree of patient’s acceptance of crime responsibility were significant predictors of violent recidivism. Contrary to our expectations, better coping skills and more insight into risky behaviors that can lead to relapse also contributed significantly to an increased likelihood of violent recidivism. Intellectual ability had no significant moderating effect on the associations between the factors and violent recidivism. The results offer an insight into which factors need to be prioritized during treatment.

Goals of the criminal justice system include crime control, reduction of recidivism among convicts (Heffernan & Ward, 2019), and safe reintegration of individuals discharged from high-secure forensic facilities. In the Netherlands, individuals who committed violent crimes under the influence of a severe mental illness, personality disorder (PD), or deficits in cognitive development are admitted for treatment to a high-secure forensic psychiatric center (FPC) by means of the Entrustment Act (in Dutch: TBS; Bogaerts et al., 2018). This measure can be imposed by the criminal court on mentally disordered offenders who are not or only partially held accountable for their offenses and who are considered dangerous to society without treatment. A total lack of accountability implies immediate TBS treatment, while partial accountability leads to imprisonment preceding mandatory treatment within the FPC (Van der Linde et al., 2020). Research has shown recidivism rates of 19% to 23% within 2 years after expiration of the TBS measure (Drieschner et al., 2018). The negative impact of recidivism on victims, society, and offenders highlights the importance of reducing recidivism among forensic psychiatric patients (Nagtegaal et al., 2016). Reduction in recidivism is linked to the Risk-Need-Responsivity (RNR) model (Bonta & Andrews, 2007), which originated from general personality and cognitive social learning perspectives. According to this model, treatment should address the criminogenic needs of high-risk individuals, such as antisocial behavior and hostility. Interventions must be theoretically substantiated and provide evidence of effectiveness and should be adapted to the offender’s personal characteristics (e.g., motivation). According to the RNR model, risk factors (RFs) can be divided into two categories: static and dynamic RFs (Bonta & Andrews, 2007). Static factors are aspects of the offender’s history but unchangeable (e.g., family background, criminal history, age at first conviction; Probst et al., 2020; Spreen et al., 2014). They are good predictors but lack the ability to monitor changes in risk and treatment effects (Heffernan et al., 2019). In contrast, dynamic (clinical) RFs are changeable aspects of individuals and their environments that are expected to increase the likelihood of recidivism (Mann et al., 2010). The assessment of dynamic RFs is essential to forensic correctional practice as it can help set targets for interventions to reduce the risk of recidivism and provide insight into treatment progress (Klepfisz et al., 2016). In the Netherlands, the assessment of these dynamic RFs for forensic psychiatric patients must be performed at least once a year (Bogaerts et al., 2020). One of the most commonly used tools for this assessment is the Historical, Clinical, Future (Historisch Klinisch Toekomst)-Revised (HKT-R; Spreen et al., 2014). This study will focus on the dynamic (clinical) factors included in the HKT-R (see Table 1).

**Table 1.** Risk and Protective Factors of the Clinical Domain of the HKT-R.

Risk factors	Protective factors
Antisocial behavior	Self-reliance
Hostility	Cooperation with treatment
Impulsivity	Labor skills
Violation of terms and agreements	Social skills
Addiction	Coping skills
Psychotic symptoms	Problem insight
Influence of risky network members	Responsibility for the offense

*Note.* HKT-R = Historical, Clinical, Future-Revised.

Although the RNR model is used as a guideline for treatment, there is a strong emphasis on RFs and little attention has been paid to protective factors (PFs; Bogaerts et al., 2020; Ward & Mann, 2004). Therefore, the Good Lives Model (GLM) has been developed as an alternative and enrichment to the RNR. The GLM is a more constructive, strength-based approach to offender rehabilitation that focuses on increasing competencies and skills to reduce the risk of recidivism (Ward & Mann, 2004). The components of treatment include motivating offenders for change and building therapeutic alliances (Bogaerts et al., 2020; Ward & Mann, 2004). Research on the relationships between PFs and recidivism is scarce, and PFs have been theorized more than empirically studied (Heffernan & Ward, 2017; Serin et al., 2016). While research has led to the consensus that improving both dynamic RFs and PFs during treatment are valuable indicators of treatment progress and recidivism (Bogaerts et al., 2020; De Vries Robbé et al., 2015; Janković, van Boxtel et al., 2021), little is known about which individual factors contribute more strongly to violent recidivism when controlling for the effects of other factors.

## Dynamic RFs and PFs in Relation to Violent Recidivism

There is some evidence that certain factors, such as *antisocial behavior*, might predict violent recidivism better than others. Antisocial behavior is shown when individuals pursue their own goals and pleasure without regard for the feelings and interests of others (Spreen et al., 2014). Individuals with an antisocial personality are characterized by impulsive, irresponsible, and hostile behavior (Janković, Masthoff et al., 2021), which can make them more likely to end up in violent and harmful conflict situations (Moffitt, 2018). Likewise, *hostile* individuals tend to attribute hostile motives to others, systems, or institutions, which leads to reacting angrily, believing that others are out to get them, and possible verbal and physical aggression (Spreen et al., 2014). Besides antisocial behavior and hostility, *impulsivity* is also considered a strong predictor of aggression; it features a lack of control, which may indicate a desire for immediate rewards or an inability to delay gratification. This can lead to a rapid response to provocation or frustration and can pose a risk for violence (Douglas & Skeem, 2005; Hildebrand & de Ruiter, 2012; Jankovic et al., 2021). Indeed, research has shown that impulsivity, antisocial behavior, and hostility are common in recently admitted forensic psychiatric patients (Bogaerts et al., 2020). These individuals may also be more likely to engage in institutional misconduct (e.g., prohibited possession of dangerous objects, severe verbal and/or physical aggression) which can put them at greater risk of recidivism (Bogaerts et al., 2020). Institutional misconduct (i.e., *“violation of terms”*) is not only a proxy measure of a propensity to offend (Trulson et al., 2011), but also contributes to all types of recidivism, including violent, property, and other recidivism (Cochran et al., 2014).

*Substance abuse* is another factor often present in offenders (Douglas & Skeem, 2005). Kraanen et al. (2012) found a prevalence of a substance use disorder of 61.5% among general violent offenders and 29.9% were intoxicated during the offense. Substance abuse could lead to disinhibition, making aggression more likely (Douglas & Skeem, 2005). It could also negatively affect PFs such as the ability to solve problems, potentially leading to conflict situations (Heffernan & Ward, 2017). Substance abuse is thought to be associated with violence, especially in inpatients (van der Kraan et al., 2014) and to a lesser extent in outpatients (Eisenberg et al., 2019). Furthermore, research shows that psychosis is significantly associated with a greater risk of violence (Klepfisz et al., 2016). *Psychotic symptoms* can directly increase the risk of violence when individuals act upon their symptoms (Douglas & Skeem, 2005). These symptoms can also indirectly increase the risk due to stress, frustration and agitation, for example by eliciting antisocial cognitions and negatively influencing self-reliance (Bogaerts et al., 2020; Douglas & Skeem, 2005). *Self-reliance* is an important protective factor to consider against recidivism. It refers to the patient’s ability to complete essential daily tasks independently (e.g., personal hygiene, care for his surroundings). Offenders, particularly those detained for a long time, tend to experience diminished independence. The literature suggests that rehabilitation programs should provide support to offenders to minimize deficiencies in the realm of self-reliance, as these skills are important for organizing their lives independently outside of the closed system after being released from the institution (United Nations Office on Drugs and Crime, n.d). In addition, individuals with psychotic symptoms may have low social support, while high social support can buffer against the symptoms, and thus reoffending (Douglas & Skeem, 2005; Guay et al., 2020). In contrast, a *non-supportive social network* has been shown to predict violence (Douglas & Skeem, 2005; Kaplan et al., 1987).

With regard to other PFs, having certain skills can protect against recidivism. Having a job is more likely when someone possesses *labor skills* (e.g., following instructions; Spreen et al., 2014). Yahner and Visher (2008) followed 145 men released from prisons in 2002 and 2003 up to 3 years after release. Men who had worked for at least a week had a lower predicted probability of reincarceration compared to men who had not. Obtaining and keeping suitable work reduces the risk of recidivism by providing structure, informal control, emotional support, and a legitimate source of income (Ramakers et al., 2017; Sapouna et al., 2011). Additionally, having good *social skills* can help maintain a job and provide a buffer against antisocial behavior (Bogaerts et al., 2020; Heffernan & Ward, 2017; Spreen et al., 2014). It can also lead to better and more committed relationships with pro-social network members, which, along with having a job, is more common among non-recidivists compared to recidivists (Berg & Huebner, 2011). Moreover, non-recidivists do not necessarily have fewer social problems than recidivists, but there is evidence suggesting that they are more psychologically resilient with higher levels of self-efficacy and better *coping skills* (i.e., ability to solve problems independently; Sapouna et al., 2011). Thus, it seems that labor, social and coping skills may prevent recidivism, especially in the context of RFs.

Lastly, poor *problem insight* has been associated with a lower likelihood of violent recidivism (Dowden et al., 1999), and failure to take *responsibility for the offense* is often seen in individuals with an antisocial personality (Van der Linde et al., 2020). It has been shown that patients who lack problem insight are more likely to have an impaired capability to take responsibility for the offense (Bogaerts et al., 2020) which may lead to *poorer treatment adherence*, and subsequently to recidivism. Several studies have found a direct association between denial and/or minimization and violent recidivism, however, the utility of these constructs as a predictor of sexual recidivism is still being hotly debated (Hanson & Morton-Bourgon, 2005; Marshall et al., 2011; Ware & Blagden, 2020). In sum, specific dynamic clinical factors might be more strongly predictive of violent recidivism, but extensive research is lacking, especially with regards to PFs.

## Intellectual Ability (IA), RFs and PRs, and Violent Recidivism

Despite the direct links between both RFs and PFs with violent recidivism, there are indications that these links may be modified by intellectual ability (IA). The opinion that offenders are characterized by lower levels of IA than non-offenders has been held for decades (Gendreau et al., 1996). In their systematic review, Hirschi and Hindelang (1977) showed that intelligence was associated with delinquent behavior as measured by official records and self-reports. Delinquents generally exhibit intelligence quotients (IQs) that are half a standard deviation lower than individuals from the community (Hirschi & Hindelang, 1977). Furthermore, individuals with low IA show more recidivism and recidivists score lower than first offenders (Guay et al., 2005; Richter et al., 1996). People with lower intelligence may be more prone to crime because of weaker cognitive skills, such as anticipating consequences and recognizing suffering in others (Guay et al., 2005). However, according to the differential detection hypothesis, this inverse association between IA and offending is considered spurious, meaning that IA of officially convicted offenders is not representative of IA of offender samples in general (Moffitt & Silva, 1988). In other words, less intelligent offenders are more likely to be arrested than more intelligent offenders who are somehow better to avoid being caught (Moffitt & Silva, 1988; Stark, 1975). Moreover, most previous research that indicated a negative association between IA and (re)offending assumed a linear association. Several researchers have suggested that this association is curvilinear, such that lower and higher levels of IA are associated with lower levels of offending (e.g., Lindsay & Taylor, 2010; Mears & Cochran, 2013). A better understanding of the association between IA and (re)offending is further limited by the fact that studies vary widely in how they measure intelligence, with some using binary coding, quartiles, deciles or continuous measures. To address this gap in the IA and (re)offending literature, it has therefore been suggested to consider the entire range of IA and to examine IA as a continuous measure (Mears & Cochran, 2013).

Furthermore, intelligence may be indirectly linked to recidivism through its effect on factors such as school performance and pro-social success opportunities (Hirschi & Hindelang, 1977). For example, lower IA leads to poor school performance and less successful negotiation in social relationships and situations. This may lead to a greater association with delinquent peers and an increased risk of reoffending (Mears & Cochran, 2013). Offenders with lower IA also exhibit higher treatment drop-out rates, which may be due to rigid thinking or the lack of required information processing skills and learning abilities that are essential to treatment (Klein Tuente et al., 2019; Newberry & Shuker, 2011). Moreover, offenders with low IA were more likely to attribute responsibility for their offenses to external circumstances (Newberry & Shuker, 2011), consistent with an external locus of control present in individuals with intellectual disability (ID; Wehmeyer & Palmer, 1997). ID is characterized by deficits in general mental abilities, such as reasoning, problem solving, judgment, and learning from experience (American Psychiatric Association [APA], 2013). A substantial part of the ID population shows more anger and aggression, putting them at risk for delinquent behavior (Asscher et al., 2012). However, not all studies find consistent results. Hassiotis et al. (2011) found a higher risk of psychosis in ID offenders compared to non-ID offenders, whereas Vinkers (2013) found that ID offenders suffered less from psychotic disorders. This may be due to the definition of psychosis (i.e., based on lay interviews or clinical examination). Furthermore, it appears that offenders with ID show less substance abuse (Taggart et al., 2006; Vinkers, 2013), while Hassiotis et al. (2008) found that there was more alcohol abuse and more severe alcohol dependence in the borderline IA group (i.e., IQ between 70 and 84), compared to the normal IA group. With regards to PFs, forensic ID patients have difficulties solving social problems (Lindsay & Taylor, 2008) and finding permanent employment (Vinkers, 2013). It is also believed that individuals with ID have poorer coping strategies, which may increase the negative influence of RFs. Thus, based on existing literature, it could be suggested that behavioral problems are more present in individuals with lower IA, where they lack the needed PFs to cope with their RFs.

To summarize, more research is necessary to expand our understanding of how the entire range of IA affects reoffending in forensic psychiatric patients. In addition, since some researchers have documented a curvilinear association between IA and (re)offending, it can also be assumed that the predictive value of RFs and PRs in reoffending may vary at different levels of IA. However, to our knowledge, no previous research has investigated whether IA can moderate the association of RFs and PFs with violent recidivism.

## The Present Study

The goal of this study was to investigate which individual dynamic RFs and PFs based on the 14 clinical indicators of the HKT-R, are the best predictors of violent recidivism in forensic psychiatric patients up to 5 years after their unconditional release from FPCs. We first hypothesized that, among RFs, antisocial behavior, hostility, impulsivity, and violation of terms and agreements would be better predictors of violent recidivism, than addiction, psychotic symptoms, and influence of risky network members (H1). Second, among PFs, it is assumed that responsibility for the offense, problem insight, and labor, social and coping skills reduce violent recidivism more than self-reliance and cooperation with treatment (H2). In addition, we have investigated whether IA can moderate the relationships between the 14 HKT-R factors and violent recidivism. Not much can be speculated about the moderating role of IA in these relationships. However, there is some indirect evidence to suggest that IA may moderate the link between certain HKT-R factors and recidivism. We hypothesized that a higher IA would diminish the positive relationship between the RFs violation of terms and agreements, hostility, and violent recidivism (H3). We further hypothesized that a higher IA would enhance the negative relationship between the PFs responsibility for the offense and social, labor, and coping skills, and violent recidivism (H4).

## Methods

### Participants and Procedure

The sample consisted of 347 forensic psychiatric patients who were unconditionally released between 2004 and 2008 from any of the 12 Dutch FPCs.^1^ Of these 347 patients, 317 were male and 30 were female. Given the low number of female patients and the fact that the HKT-R has not been validated for female offenders (Spreen et al., 2014), we decided to exclude them from this study. Information was collected for all participants on demographic characteristics, psychiatric disorders according to the *Diagnostic and Statistical Manual of Mental Disorders* (4th ed., text rev. [DSM-IV-TR]; APA, 2000), intellectual ability, and criminal records through electronic patient files. In addition, 20 trained coders assessed the HKT-R using information from the patients’ criminal files, with detailed descriptions of their background and criminal history, psychiatric evaluation reports, treatment plans, leave requests, and prolongation advice. Bogaerts et al. (2018) assessed the interrater reliability in a sample of 347 patients who were discharged between 2004 and 2008 from the 12 FPCs, by calculating the intraclass correlation coefficient (ICC). The Clinical domain had good interrater reliability with the ICC being .85 with a 95% confidence interval ranging from .67 to .94. This study used scores at the time of unconditional release as offenders are at that moment considered as having a low enough risk for recidivism to enter society and are no longer supervised by correctional services (Van der Linde et al., 2020). All data were anonymized before running analyses. In this study, patients were not able to give informed consent because many of them no longer reside in the institutions and a number of them have already died. However, in exceptional cases, like in this one, research with patient file data is possible without permission (Article 7:458 paragraph 3 of the Dutch Civil Code [in Dutch: BW]). Furthermore, this research serves the public interest, namely the safety of society and the study with this large group of patients cannot be performed in any other way (as mentioned in Uitzondering op toestemmingsvereiste [Exception to consent requirement; Art. 7: 458 BW]). The study was approved by the ethical review board of Tilburg University School of Social and Behavioral Sciences and preregistered at AsPredicted.^2^

### Measures

#### Historical Clinical Future-Revised

The HKT-R is a structured professional risk assessment instrument that assesses 12 Historical, 14 Clinical, and seven Future indicators for violent reoffending in forensic psychiatric patients (Spreen et al., 2014). The Historical domain refers to a patients’ history up to the moment of arrest for the current TBS-index offense. The Clinical domain refers to the patients’ behavior during the 12 months preceding the moment of risk assessment. The Future domain refers to the risk estimation in situations as transfer to and extension of leave of absence, transfer to a subsequent institution, and/or when a patient directly enters society without supervision (Spreen et al., 2014). Only the 14 clinical indicators equally divided into seven risk and seven protective factors (see Table 1) were used in the current study because these are changeable by treatment. All factors were rated on a 5-point scale, ranging from 0 = *none or very low risk* to 4 = *high level of risk*. For this study, the data for the PFs were recoded, ranging from 0 = *no protection* to 4 = *high level of protection*. In previous research, the predictive validity was assessed marginal at both 2 years (admission: area under the curve [AUC] = .62, discharge; AUC = .63) and 5 years (admission: AUC = .69; discharge: AUC = .62; Bogaerts et al., 2018). In the current sample, the Clinical domain had a good internal consistency with Cronbach’s α = .83.

#### Wechsler Adult Intelligence Scale IV-NL (WAIS-IV-NL)

The IA of patients was assessed by a clinician using the WAIS-IV-NL at the time of admission to the FPC. The WAIS-IV-NL is the Dutch adaptation of the WAIS-IV (Wechsler, 2012). It is a comprehensive clinical instrument for assessing the IAs of older adolescents and adults (Valentine et al., 2020) The Full-Scale IQ score consists of four indexes with 10 core subtests and five supplemental tests. These are the Verbal Comprehension Index (Similarities, Vocabulary, Information, and supplemental Comprehension), Perceptual Reasoning Index (Block Design, Matrix Reasoning, Visual Puzzles, supplemental Figure Weights, and supplemental Picture Completion), Working Memory Index (Digit Span, Arithmetic, and supplemental Letter-Number Sequencing), and Processing Speed Index (Symbol Search, Coding, and supplemental Cancelation; Valentine et al., 2020). The test takes up to an hour to 1.5 hours, which may be longer in certain clinical populations. The raw scores for the subtests are computed into comparable scale scores (Bastiaens et al., 2013). The split half reliability of the Full-Scale IQ score is good with an average alpha of α = .97, while the test-retest correlations are also good with a range from *r = *.94 to .96 across three age groups (Pearson, 2012). The Full-Scale IQ score was used in the current study.

#### Violent recidivism

The Dutch Ministry of Security and Justice collected official data on reconvictions after release up until July 11, 2011 that were provided to the researchers for the validation of the HKT-R (Spreen et al., 2014). For the purpose of the study, violent recidivism refers to all offenses in which violence or the threat of violence toward a person was used, including mild to moderate violence and possession of arms, power by force, severe violence, moral offenses with adults as victims, manslaughter, arson, and premeditated murder. Violent recidivism was operationalized as any new conviction for any of these offenses up to 5 years following unconditional release and coded with 0 = *non-recidivist* and 1 = *violent recidivist*.

### Statistical Analyses

To analyze the data, we used IBM SPSS Statistics version 23 and the Process extension (Hayes, 2017). First, we examined the presence of outliers and missing values. One outlier was identified with regards to IA, using Mahalanobis distance, Cook’s distance and leverage values and was therefore excluded from the sample. Following, one participant with more than 10% missing values was detected and excluded from the sample (Bennett, 2001), leaving a total of 315 participants. In addition, we performed the Little’s (1988) Missing Completely at Random (MCAR) test to check if other missing data were completely at random. The other missing data were not completely randomly missing with χ^2^(1036) = 1181.09, *p* = .001. It should be noted that the HKT-R factors were scored by trained researchers, not by self-report and hence it could be assumed that data were not completely randomly missing probably due to insufficient information in the electronic patient files, rather than specific patterns in the missing data values. Therefore, the remaining missing data were multiple imputed (Royston, 2004). The multiple imputation method uses information from other variables in a dataset to predict and impute the missing data. It aims to allow for the uncertainty of the missing data by calculating different options (“imputations”) and combining them appropriately to make the “best” values (see Sterne et al., 2009, for more detail). Subsequently, descriptive statistics for all study variables were computed. Differences in sample characteristics between recidivists and non-recidivists were evaluated using the Chi-square test for categorical variables and Mann-Whitney U tests for ordinal or not normally distributed continuous variables. In addition, a point-biserial correlation analysis was conducted to analyze the associations between continuous indicators (i.e., the HKT-R factors and IA) and violent recidivism (i.e., the binary outcome variable). The following variables were used to answer the research question. First, the ordinal independent variables were the seven RFs and seven PFs, the dichotomous dependent variable was whether someone commits violent recidivism, and the independent continuous moderator variable was the Full-Scale IQ score.

Furthermore, to investigate how well the 14 individual HKT-R dynamic factors predict violent recidivism, a binary logistic regression was applied. A recommended “rule of thumb” in logistic regression to complete the power calculation is a minimum of 15 cases per independent variable (Khaing, 2019), which was achieved in our study (15 × 15 = 225). Next, prior to analysis, the assumptions for binary logistic regression were checked. The first assumption of a dichotomous dependent variable was met. With regards to the second assumption of one or more independent variables measured on a continuous or nominal scale, 14 of the 15 independent variables were ordinal, but these can also be treated as continuous variables in binary logistic regression. The third assumption of independence of observations and mutually exclusiveness and exhaustiveness of the dependent variable and the independent variables was met. Moreover, the linearity of the continuous variables with respect to the logit of the dependent variable was assessed via the Box and Tidwell (1962) procedure. A Bonferroni correction was applied resulting in statistical significance being accepted when *p* < .0016 (Tabachnick & Fidell, 2014). All continuous independent variables were found to be linearly related to the logit of the dependent variable. Next, by using Tolerance/Variance Inflation factor (VIF) values we identified no multicollinearity with respect to the independent variables. Given that the data met the assumptions, binary logistic regression was performed with seven RFs, and seven PFs as predictors of violent recidivism. Finally, we performed moderation analyses using PROCESS (model 1). According to Baron and Kenny (1986) the function of a moderation effect is to indicate under which specific condition a predictor is related to a dependent variable. It could have different effects on the outcome variable: it could amplify, dampen or reverse it. PROCESS was performed with all 14 factors separately as independent variables, IA as moderator and violent recidivism as dependent variable, while the two-way interaction estimates were generated automatically. All other RFs and PFs were included as covariates to reveal unique effects. In the case of significant moderation effects, the nature of these interactions would be further explored by conducting simple effect analyses.

## Results

The final sample consisted of 315 male forensic psychiatric patients. The majority (95.2%) had a Dutch nationality (*n = *300), 2.9% had another nationality than Dutch (*n = *9) and 1.9% had an additional nationality (*n = *6). The majority (80%) received both TBS and a prison sentence (*n = *252) and the remaining 20% were placed directly in an FPC. The age at admission ranged from 17 to 66 years (*M = *31.8, *SD* = 8.69). Previously committed offenses in order of frequency were fiscal capital and profit offenses (*n = *203, 64.4%), mild to moderate violence and possession of arms (*n = *191, 60.6%), traffic violations and civil disorder (*n = *130, 41.3%), power by force (*n = *124, 39.4%), manslaughter (*n = *120, 38.1%), destruction of property (*n = *106, 33.7%), severe violence (*n = *85, 27%), moral offenses (*n = *56, 17.8%), premeditated murder (*n* = 52, 16.5%), arson (*n* = 43, 13.7%), drug-related offenses (*n = *24, 7.6%), and moral offenses with minors as victims (*n = *23, 7.3%). The most common psychiatric disorders according to the DSM-IV-TR (APA, 2000) were personality disorder not otherwise specified (*n = *165, 52.4%), substance related disorder (*n = *111, 35.2%), cluster B personality disorders (*n = *83, 26.3%), and schizophrenia and other psychotic disorders (*n = *70, 22.2%; see Table 2). The mean IA in this sample was 98 (range 52–139, *SD* = 15.23). Fifteen participants (4.8%) met the DSM-IV-TR criteria for ID. A total of 55 participants (17.5%) committed violent recidivism within 5 years. The descriptive statistics of the clinical factors are presented in Table 3.

**Table 2.** Axis I and Axis II Psychiatric Disorders.

Variable	Entire sample	Violent recidivists	Non-recidivists	χ^2^	*p*
	*N* (%)	*N* (%)	*N* (%)		
Axis I					
Disorders usually first diagnosed in infancy, childhood, or adolescence	15 (4.8)	5 (9.1)	10 (3.8)	2.75	.152
Substance-related disorders	111 (35.2)	22 (44.0)	89 (34.2)	0.66	.439
Schizophrenia and other psychotic disorders	70 (22.2)	10 (18.2)	60 (23.1)	0.63	.480
Mood disorders	20 (6.3)	1 (1.8)	19 (7.3)	2.30	.219
Anxiety disorders	11 (3.5)	3 (5.5)	8 (3.1)	0.76	.414
Sexual and gender identity disorders	12 (3.8)	1 (1.8)	11 (4.2)	0.72	.699
Impulse-control disorders not elsewhere classified	11 (3.5)	1 (1.8)	10 (3.8)	0.55	.696
Axis II					
Intellectual disability	11 (3.5)	1 (1.8)	14 (5.4)	1.27	.483
Cluster A personality disorder	83 (26.3)	3 (5.5)	8 (3.1)	0.76	.414
Cluster B personality disorder	10 (3.2)	21 (38.2)	62 (23.8)	4.81	.042^*^
Cluster C personality disorders	165 (52.4)	0 (0.0)	10 (3.8)	2.12	.220
Personality disorder not otherwise specified	15 (4.8)	29 (52.7)	136 (52.3)	<0.00	1.00

*Note*. Only the most frequent Axis I diagnoses are presented in the table; Chi-square was applied to evaluate differences between violent recidivists and non-recidivists.

* *p* < .05.

**Table 3.** Descriptive Statistics of the Clinical Factors for Violent Recidivists and Non-Recidivists.

Clinical domain	Entire sample				Violent recidivists				Non-recidivists			
	Min	Max	*M*	*SD*	Min	Max	*M*	*SD*	Min	Max	*M*	*SD*
Risk factors												
Antisocial behavior	0	4	0.5	0.9	0	4	1.0	1.2	0	4	0.4	0.8
Hostility	0	4	0.5	0.8	0	4	1.2	1.0	0	3	0.4	0.7
Impulsivity	0	4	0.9	1.0	0	4	1.5	1.2	0	4	0.8	1.0
Violation of terms and agreements	0	4	0.4	1.0	0	4	0.9	1.4	0	4	0.3	0.8
Addiction	0	4	0.4	1.0	0	4	1.0	1.4	0	3	0.3	0.8
Psychotic symptoms	0	2	0.1	0.4	0	2	0.1	0.4	0	2	0.2	0.4
Influence of risky network members	0	3	0.1	0.3	0	3	0.3	0.6	0	2	0.0	0.2
Protective factors												
Self-reliance	1	4	2.8	0.7	1	4	3.7	0.7	1	4	3.7	0.7
Cooperation with treatment	0	4	3.4	1.0	0	4	2.1	1.2	0	4	3.5	0.9
Labor skills	0	4	3.4	1.0	0	4	2.7	1.3	0	4	3.6	0.8
Social skills	0	4	3.1	1.0	1	4	2.6	1.0	0	4	3.2	0.9
Coping skills	0	4	2.7	1.0	0	4	2.5	1.1	0	4	2.8	1.0
Problem insight	0	4	2.8	1.0	0	4	2.6	1.2	0	4	2.9	1.0
Responsibility for the offense	0	4	2.5	1.2	0	4	2.1	1.2	0	4	2.7	1.2

*Note.* Min = Minimum; Max = Maximum; *M* = Mean; *SD* = Standard deviation.

Moreover, there were some significant differences between violent recidivists and non-recidivists regarding psychiatric diagnoses and history of criminal offenses. Chi-Square tests (see Table 2) showed that cluster B personality disorders were more frequent in the group of violent recidivists than in the group of non-recidivists. In addition, Mann-Whitney U tests (see Table A1 in the appendix) showed that violent recidivists had significantly more offenses related to traffic violations and civil disorders, fiscal capital and profit offenses, mild to moderate violence and possession of arms, power by force, and severe violence. However, violent recidivists had significantly fewer moral offenses with minors as victims and less premeditated murder. Moreover, violent recidivists had a significantly broader history of offenses across categories than non-recidivists. Lastly, violent recidivists had a significantly lower IA than non-recidivists. The differences between violent recidivists and non-recidivists in clinical HKT-R factors are presented in Table 3.

Furthermore, a point-biserial correlation analysis (see Table A2 in the appendix) revealed that with the exception of psychotic symptoms, all RFs were significantly positively associated with violent recidivism. Regarding PFs, cooperation with treatment, labor and social skills, and responsibility for the offense were significantly negatively associated with violent recidivism. On the contrary, IA was not significantly associated with violent recidivism.

Finally, a logistic regression analysis was performed to analyze the impact of the 14 clinical factors on the risk of violent recidivism. The model containing all factors was significant, χ^2^(14) = 94.44, *p* < .001, indicating that it could distinguish between violent recidivists and non-recidivists. The Hosmer and Lemeshow test was not significant, χ^2^(8) = 6.31, *p* = .613, indicating that the data fit the model well. The model explained 42.9% (Nagelkerke *R*^2^) of the variance in violent recidivism and correctly classified 85.4% of cases. Looking at the factors separately, influence of risky network members (*OR*= 6.08), hostility (*OR* = 1.85), impulsivity (*OR* = 1.74), and addiction (*OR* = 1.42) were significantly positively associated with violent recidivism, meaning that the likelihood of violent recidivism increases when these RFs increase (see Table 4). Antisocial behavior, psychotic symptoms and violation of terms and agreements were no significant predictors. With regards to PFs, responsibility for the offense (*OR* = 0.53), labor skills (*OR* = 0.50), and social skills (*OR* = 0.46) had a significant negative association with violent recidivism, meaning that the likelihood of violent recidivism decreases when these PFs increase. In contrast, coping skills (*OR* = 2.73) and problem insight (*OR* = 1.83) had significant positive associations, meaning that the likelihood of violent recidivism increases when these PFs increase. Cooperation with treatment and self-reliance were no significant predictors.

**Table 4.** Logistic Regression Predicting the Likelihood of Violent Recidivism (*n* = 315).

Main effects	*b*	*SE*	*p*	*Exp*(*b*)	95% CI for *Exp*(*b*)	
					Lower	Upper
Risk factors						
Antisocial behavior	−.28	0.27	.303	0.76	0.45	1.28
Hostility	.62	0.29	.036	1.85	1.04	3.30
Impulsivity	.56	0.25	.028	1.74	1.06	2.86
Violation of terms and agreements	−.02	0.20	.924	0.98	0.67	1.44
Addiction	.35	0.18	.047	1.42	1.00	2.00
Psychotic symptoms	−.82	0.53	.120	0.44	0.16	1.24
Influence of risky network members	1.81	0.63	.004	6.08	1.78	20.77
Protective factors						
Self-reliance	.27	0.35	.441	1.31	0.66	2.63
Cooperation with treatment	−.15	0.25	.556	0.86	0.53	1.41
Labor skills	−.69	0.22	.002	0.50	0.33	0.77
Social skills	−.77	0.25	.002	0.46	0.29	0.76
Coping skills	1.00	0.31	.001	2.73	1.46	5.02
Problem insight	.60	0.28	.031	1.83	1.06	3.17
Responsibility for the offense	−.63	0.20	.002	0.53	0.36	0.79
Constant	−1.69	1.67	.311	0.18		

*Note. Exp*(*b*) = odds ratio.

Subsequently, it was tested whether IA moderates the association between the RFs and PFs, and violent recidivism. None of the interactions with IA were significant (see Table 5), meaning that the level of IA did not moderate the association between the 14 factors and violent recidivism. However, in all analyzed models, IA was found to have a significant direct negative effect on violent recidivism (*OR* = 0.49).

**Table 5.** Interaction Analyses Between the Clinical Factors and Intellectual Ability (IA) in Predicting the Likelihood of Violent Recidivism (*n* = 315).

	*b*	*SE*	*z*	*p*	95% CI	
					Lower	Upper
*Risk factors*						
Model 1						
Antisocial behavior	−.19	.28	−.69	.49	−.74	.35
IA	−.04	.01	−2.77	.01	−.07	−.01
Antisocial behavior × IA	.03	.02	1.70	.09	-.01	.06
Model 2						
Hostility	.70	.31	2.29	.02	.10	1.30
IA	−.04	.01	−2.80	.01	−.07	−.01
Hostility × IA	.01	.02	.47	.64	−.03	.04
Model 3						
Impulsivity	.64	.27	2.40	.02	.12	1.17
IA	−.04	.01	−2.84	.01	−.07	−.01
Impulsivity × IA	.02	.01	1.30	.19	−.01	.04
Model 4						
Violation of terms and agreements	−.04	.20	−.18	.86	−.44	.36
IA	−.04	.01	−2.80	.01	−.07	−.01
Violation of terms and agreements × IA	−.01	.02	−.66	.51	−.05	.02
Model 5						
Addiction	.36	.18	2.01	.04	.01	.72
IA	−.04	.01	−2.75	.01	−.07	−.01
Addiction × IA	.01	.01	.60	.55	−.02	.04
Model 6						
Psychotic symptoms	−.73	.57	−1.27	.20	−1.85	.39
IA	−.04	.02	−2.76	.01	−.07	−.01
Psychotic symptoms × IA	−.03	.06	−.61	.54	−.14	.08
Model 7						
Influence of risky network members	1.82	.74	2.47	.01	.38	3.27
IA	−.04	.02	−2.74	.01	−.07	−.01
Influence of risky network members × IA	−.01	.05	−.20	.84	−.10	.08
*Protective factors*						
Model 8						
Self-reliance	.22	.39	.56	.58	−.55	.99
IA	−.04	.02	−2.47	.01	−.06	−.01
Self-reliance × IA	−.04	.03	−1.45	.15	−.09	.01
Model 9						
Cooperation with treatment	−.27	.26	−1.02	.31	-. 77	.24
IA	−.04	.02	−2.47	.01	−.06	−.01
Cooperation with treatment × IA	−.01	.02	−.43	.67	−.04	.03
Model 10						
Labor skills	−.79	.24	−3.33	.01	−1.25	−.32
IA	−.04	.02	−2.80	.01	−.06	−.01
Labor skills × IA	.02	.02	1.09	.28	−.02	.06
Model 11						
Social skills	−.71	.25	−2.80	.01	−1.20	−.21
IA	−.04	.02	−2.70	.01	−.06	−.01
Social skills × IA	.01	.01	.03	.98	−.02	.03
Model 12						
Coping skills	1.09	.33	3.36	.01	.45	1.73
IA	−.04	.02	−2.59	.01	−.07	−.01
Coping skills × IA	−.01	.02	−.65	.52	−.04	.02
Model 13						
Problem insight	.70	.29	2.41	.02	.13	1.27
IA	−.04	.02	−2.59	.01	−.06	−.01
Problem insight × IA	−.01	.01	−.40	.69	−.04	.02
Model 14						
Responsibility for the offense	−.60	.21	−2.85	.01	−1.02	−.19
IA	−.04	.02	−2.49	.01	−.06	−.01
Responsibility for the offense × IA	.01	.01	1.03	.30	−.01	.04

*Note.* Fourteen separate interaction analyses were performed in which one factor was entered as the predictor and the remaining 13 factors were entered as covariates.

## Discussion

This was the first study that investigated which dynamic RFs and PFs, based on the 14 clinical factors of the HKT-R, significantly predicted violent recidivism in a sample of male forensic psychiatric patients up to 5 years after unconditional release from any of the 12 Dutch FPCs. In addition, we investigated whether these associations were influenced by IA. The results showed that certain individual RFs and PFs are indeed significant predictors of violent recidivism when controlling for the influence of the other factors in the model. However, there were some deviations from the expected direction. Similarly, contrary to our expectations, we did not find that IA significantly moderated the association of the individual RFs and PFs with violent recidivism. However, IA directly and negatively predicted violent recidivism.

Our first hypothesis which states that antisocial behavior, hostility, impulsivity, and violations of terms and agreements would contribute more strongly to violent recidivism than addiction, psychotic symptoms and influence of risky network members, is partially accepted. As expected, patients who were characterized by higher levels of impulsivity and hostility at the time of unconditional release were more likely to violently reoffend up to 5 years from release, which is in line with previous research (Douglas & Skeem, 2005; Spreen et al., 2014). However, in previous studies, the link between hostility and recidivism was mainly determined in a sample of sexual offenders (Firestone et al., 2005; Pettersen et al., 2015), while the current study demonstrates this relationship also in a sample of violent offenders. Moreover, the results showed that influence of risky network members is the strongest predictor of violent recidivism. Although we expected that this association would be less strong, it is in line with previous research showing that social support for crime based on criminal friends and isolation from prosocial others, is one of the Central Eight (i.e., major risk factors for recidivism) according to the RNR model (Bonta & Andrews, 2007). Our finding adds to existing studies in which they focus more on the impact of the presence or lack of social support and prosocial networks, rather than a negative influence of a risky network (Douglas & Skeem, 2005; Guay et al., 2020). Furthermore, consistent with previous studies, we found that patients with higher levels of substance abuse were more likely to recidivate (Doyle, 2012; Langan & Levin, 2002; Mannerfelt & Håkansson, 2018).

Contrary to our expectations, the other three RFs—namely antisocial behavior, psychotic symptoms and violation of terms and agreements—were no significant predictors of violent recidivism when entered alongside other RFs and PFs. Although antisocial behavior had a strong positive bivariate correlation with violent recidivism, this association became insignificant in the presence of other factors probably due to the shared variance with these factors, especially with hostility and impulsivity. Furthermore, unexpectedly, psychotic symptoms did not significantly predict violent reoffending. However, in the systematic literature review of Lamberti et al. (2020), no clear consensus has been highlighted in existing research as well. Specifically, 14 out of 50 studies did not find an association between psychotic symptoms and criminal recidivism and no clear conclusions could be made; the studies differed widely in their methods and did not take the same covariates into account. Another possible factor that could play a role in the lack of association is the fact that the maximum score for psychotic symptoms in this sample was equal to 2. This means that the psychotic symptoms did only lead to undirected transboundary behavior, and no directed severe aggressive behavior. It could be also speculated that some (ex-) patients who stopped taking antipsychotics after release, may run into mental health care more quickly due to a psychiatric dysregulation, leaving them less time to recidivate. In contrast, the personality-impaired person may be less likely to stand out and have all the time and opportunity for a criminal offense. However, there are no data on the development of psychotic symptoms after unconditional release and thus it could be just a speculation. Lastly, despite a significant bivariate association between violation of terms and agreements and violent recidivism, this association was not significant when other RFs and PFs were entered into the model. Since violation of terms and agreements had a moderate to large positive association with both impulsivity and hostility and a negative moderate to large association with cooperation with treatment, it could be that the effect of violation of terms and agreements on violent recidivism is accounted for these well-established factors. Indeed, past research has shown that individuals who violate terms and agreements are more likely to be impulsive, which in turn can lead to aggression and non-compliance with treatment (APA, 2000; Bresin, 2019; Young et al., 2018).

Additional research is needed to investigate whether impulsivity, hostility and cooperation with treatment may mediate the association between violation of terms and agreement and violent recidivism.

Our second hypothesis which states that responsibility for the offense, problem insight, and labor, social, and coping skills, would more strongly reduce violent recidivism than self-reliance and cooperation with treatment is also partially accepted. Results showed that patients who scored higher on labor and social skills at time of unconditional release, were less likely to violently reoffend within 5 years. This is in line with our expectations based on previous research in which possessing these skills was associated with less chance of reoffending (Berg & Huebner, 2011; Sapouna et al., 2011). Moreover, the results indicate that patients who are more likely to accept responsibility for the committed offense are less likely to recidivate. Tangney et al. (2014) showed that experiencing guilt negatively predicted criminal recidivism within a 1-year follow-up. This suggests that there may be a role of the negative emotions accompanying the feeling of responsibility.

Surprisingly, our results show that having more problem insight leads to higher odds of violent recidivism. This is not in line with our expectations nor previous findings concerning violent juvenile recidivists who scored higher on lack of problem insight (Mulder et al., 2010). Likewise, we found a significant positive relationship between coping skills and violent reoffending. These results could possibly be explained by the environment of participants at the time of coding. Patients in an FPC live in a structured and predictable facility in which there is a surmountable amount of strain. It is suggested that although emotional coping techniques can be seen as an adequate form of noncriminal nature, they may be less effective when dealing with severe strain (Agnew, 2001). Re-entering society is accompanied by high strain, for which the emotional techniques might not be effective, which in turn could lead to increased levels of anger and violence (LaCourse et al., 2019). As such, Phillips and Lindsay (2011) found that previously incarcerated individuals used more emotion-focused and avoidance coping compared to problem-focused coping according to a coping inventory at the time of incarceration. However, during the re-entry into society, they were not able to apply the same techniques to reduce the emotional reactions to the problems they encountered, and they recidivated. Given that in the current study we made no distinction between different coping strategies, additional research is needed to investigate how differences in coping strategies are associated with violent reoffending.

Lastly, although we expected at least small negative relationships between both self-reliance and cooperation with treatment, and violent recidivism, there were no significant relationships in this study. Similar to antisocial behavior, it could be that these factors only contribute to the predictability of the entire Clinical domain given the bivariate correlations with other factors, but that they are not predictive on their own. Cooperation with treatment was indeed negatively associated with violent recidivism, but this association was no longer significant when other factors were entered into the model.

Taken together, these findings indicate that certain RFs and PFs predict violent recidivism, even when the effects of other factors in the model are taken into account. However, most significant RFs have larger effects (i.e., OR) on violent recidivism than the significant PFs. These findings somewhat favor the RNR model (Bonta & Andrews, 2007) over the GLM (Ward & Mann, 2004), but also suggest that these two models should be seen as complementary rather than contradictory.

Furthermore, we found that IA did not influence the relationships between the dynamic factors and violent recidivism. Therefore, the third and fourth hypotheses can be rejected. Although previous studies showed that a substantial part of individuals with ID score higher on certain RFs and lower on certain PFs (Asscher et al., 2012; Lindsay & Taylor, 2008; Newberry & Shuker, 2011; Vinkers, 2013), our results did not find a significant influence of the entire range of IA on the relationships between the dynamic factors and violent recidivism. Similar to violation of terms and agreements, it could be that the level of IA has a greater impact on patients’ responsiveness to the treatment according to the responsivity principle of the RNR model (Bonta & Andrews, 2007). This principle states that treatment and intervention should be adapted to the learning style, motivation, abilities and strengths of the offender, which could differ for individuals with different levels of IA. Furthermore, not taking education level into account could have played a role, given that research proposed that intelligence is indirectly linked to reoffending through school performance (Hirschi & Hindelang, 1977; Mears & Cochran, 2013). It would be interesting for future studies to investigate whether the moderation is present when education level is added as a covariate. Alternatively, it could be that we did not find a moderating effect of IA because we took into account only a full-scale IQ score, rather than specific intellectual abilities, such as verbal and performance IQs. For example, research shows that performance IQ is greater than verbal IQ in a forensic sample (Dewolfe & Ryan, 1984). Future research needs to investigate if performance and verbal IQ scores modify the association between the clinical HKT-R factors and violent recidivism. However, in all tested models, IA had a significant direct and negative effect on violent recidivism. This means that patients with higher levels of IA were less likely to reoffend violently after discharge from the FPC. Our finding corresponds with previous studies indicating that individuals with lower intelligence are more prone to crime and recidivism (e.g., Gendreau et al., 1996; Guay et al., 2005; Richter et al., 1996).

The current study had several limitations which need to be taken into consideration. First, this study used retrospective data which was collected based on patients’ criminal files. Even though extensive information was available, some of the factors could not be coded based on this information which meant these missing values had to be imputed. In addition, an assessment of the HKT-R on direct behavioral observations rather than retrospective coding would provide more accurate data. Second, this study only included males, which means that the results are only generalizable to male psychiatric forensic patients. Third, the data used in this study consisted of patients released from FPCs between 2004 and 2008, and only 55 out of 315 participants committed violent recidivism up to 5 years after unconditional release. Future studies investigating the association between the individual RFs and PFs and violent recidivism could take longer follow-up periods into account. In addition, we could not control for the time at risk because we did not have any information about whether and for what period patients were institutionalized after unconditional release. It is possible that some patients have been detained again or admitted to a (forensic) institute due to recidivism or other mental health reasons and hence did not have access to reoffend the entire 5-year follow-up period. Fourth, this study did not make a distinction between the different violent offenses, nor did it take the index offense into account. This could possibly play a role in the results, given that Drieschner et al. (2018) showed there was a higher risk of recidivism after TBS when the index offense was fiscal capital and profit offenses, and when the offender had multiple previous offenses. Fifth, the results may not be generalizable to other international forensic samples due to differences in sentencing and the Dutch nature of the HKT-R. Regarding differences in sentencing, in the United States, a substantial part of our sample would have likely been sent to prison, instead of an FPC based on a TBS-order (De Ruiter & Trestman, 2007). This could also explain why in our sample the mean score of IA was somewhat higher than the mean score of IA in forensic samples as reported in the literature and was more similar to the IA distribution of the general population. Therefore, caution should be taken when interpreting the results of this study because the results could differ in different forensic samples or similar samples with different IA distributions. Lastly, there were more covariates we could have taken into account, such as education level, age at offense, and age at release from TBS (Drieschner et al., 2018).

Despite the limitations, the current study is very important given the potential harm caused by violent recidivism to the victim, the offending individual, and society (Nagtegaal et al., 2016). This study provides contributions to both the scientific and clinical field, by highlighting that both RFs and PFs are associated with violent recidivism. It is the first study that examined how all 14 clinical factors individually contributed to violent recidivism. Additionally, it is one of the few studies that equally focuses on both RFs and PFs and the entire range of IA. Given the fact that these dynamic factors change over time and inform treatment, our findings suggest that treatment should at least be focused on the significant factors in order to reduce the chance of reoffending. Specifically, in line with the RNR model, impulsivity and hostility should be prioritized in treatment, by which it is likely that the amount of antisocial behavior will indirectly reduce too. Furthermore, our study shows that treatment should not only focus on RFs from the individual him/herself, but also on the influence of others on that individual. Additionally, treatment should target the addictions of individuals, given that in addition to social support for crime, substance abuse is part of the Central Eight (Bonta & Andrews, 2007). Moreover, according to the GLM, the chance of reoffending can be reduced by targeting the strengths of individuals (Ward & Mann, 2004). Therefore, interventions can be used to enhance the presence of labor and social skills and teach individuals how to use these skills in daily life during and after resocialization. Lastly, treatment should focus on which feelings and thoughts individuals have regarding the offense, to enhance a feeling of responsibility and reduce the chance of reoffending.

In conclusion, this study provides support for both the RNR model and the GLM; the focus should be on the needs of individuals that lead to crimes, as well as on the strengths of these individuals to divert them from committing crimes.

## Appendix

**Table A1.** Significant Differences in History of Offenses and Intellectual Ability (IA) Between Violent Recidivists and Non-Recidivists.

Variable	*U*	*Z*	*p*	Violent recidivists		Non-recidivists	
				*Md*	*M* (*SD*)	*Md*	*M* (*SD*)
Traffic violations and civil disorders	5,856.50	−2.37	.018	1	2.7 (6.1)	0	1.2 (2.9)
Fiscal and capital and profit offenses	5,347.00	−3.01	.003	4	11.8 (22.7)	2	6.4 (17.2)
Mild to moderate violence and possession of arms	5,247.50	−3.22	.001	2	3.3 (5.4)	1	2.0 (3.1)
Power by force	5,527.50	−3.01	.003	1	1.5 (1.8)	0	1.1 (2.1)
Severe violence	6,188.00	−2.02	.044	0	0.6 (1.2)	0	0.4 (0.9)
Moral offenses with minors as victims	6,517.50	−2.29	.022	0	0.0 (0.0)	0	0.5 (2.3)
Premeditated murder	6,177.50	−2.46	.014	0	0.1 (0.2)	0	0.2 (0.5)
History of offenses across categories	5,089.00	−3.40	.001	5	4.4 (1.6)	3	3.5 (1.9)
IA	5,775.00	−2.24	.025	96	94.9 (11.8)	100	98.9 (15.8)

**Table A2.** Point-Biserial Correlations Between the Main Variables.

	1	2	3	4	5	6	7	8	9	10	11	12	13	14	15	16
1. Psychotic symptoms	—															
2. Addiction	.19^**^	—														
3. Impulsivity	.11	.32^**^	—													
4. Antisocial behavior	.11^*^	.19^**^	.55^**^	—												
5. Hostility	.18^**^	.26^**^	.52^**^	.53^**^	—											
6. Violation of terms and agreements	.09	.33^**^	.41^**^	.29^**^	.44^**^	—										
7. Influence of risky network members	.13^*^	.14^*^	.19^**^	.34^**^	.32^**^	.12^*^	—									
8. Problem insight	−.29^**^	−.11	−.30^**^	−.41^**^	−.36^**^	−.20^**^	−.14^*^	—								
9. Social skills	−.19^**^	−.13^*^	−.28^**^	−.41^**^	−.45^**^	−.19^**^	−.14^*^	.49^**^	—							
10. Self-reliance	−.42^**^	−.07	−.15^**^	−.17^**^	−.18^**^	−.07	−.02	.41^**^	.29^**^	—						
11. Cooperation with treatment	−.17^**^	−.24^**^	−.49^**^	−.49^**^	−.50^**^	−.42^**^	−.28^**^	.51^**^	.39^**^	.24^**^	—					
12. Responsibility for the offense	−.13^*^	−.10	−.15^**^	−.25^**^	−.27^**^	−.20^**^	.01	.52^**^	.26^**^	.23^**^	.28^**^	—				
13. Coping skills	−.27^**^	−.18^**^	−.46^**^	−.41^**^	−.38^**^	−.27^**^	−.16^**^	.53^**^	.56^**^	.46^**^	.47^**^	.34^**^	—			
14. Labor skills	−.19^**^	−.28^**^	−.31^**^	−.42^**^	−.41^**^	−.33^**^	−.22^**^	.37^**^	.44^**^	.38^**^	.48^**^	.30^**^	.48^**^	—		
15. IA	−.07	.04	−.03	−.07	−.03	.04	.01	.28^**^	.20^**^	.28^**^	−.01	.17^**^	.14^*^	.03	—	
16. Violent recidivism	−.02	.25^**^	.25^**^	.25^**^	.39^**^	.25^**^	.27^**^	−.11	−.27^**^	.01	−.27^**^	−.19^**^	−.09	−.34^**^	−.10	—

*Note*. IA = intellectual ability.

* *p* < .05; ***p* < .01.

## Notes

1. Of these 12 institutions, six are TBS clinics, five are forensic psychiatric clinics, and one is a center for transcultural psychiatry; all have a (very) high security level and provide intensive treatment.

2.
https://aspredicted.org/CN7_FPK
